# Long-term antibiotic exposure landscapes and resistant *Escherichia coli* colonization in a densely populated setting

**DOI:** 10.1371/journal.pone.0302521

**Published:** 2024-07-09

**Authors:** Eric Ng’eno, Marlon E. Cobos, Samuel Kiplangat, Robert Mugoh, Alice Ouma, Godfrey Bigogo, Sylvia Omulo, A. Townsend Peterson

**Affiliations:** 1 Centre for Global Health Research, Kenya Medical Research Institute, Nairobi, Kenya; 2 Department of Ecology and Evolutionary Biology and Biodiversity Institute, University of Kansas, Lawrence, KS, United States of America; 3 Washington State University Global Health–Kenya, Nairobi, Kenya; 4 Paul G. Allen School for Global Health, Washington State University, Pullman, WA, United States of America; 5 University of Nairobi Institute of Tropical and Infectious Diseases, Nairobi, Kenya; North Carolina State University, UNITED STATES

## Abstract

Antibiotic exposure is associated with resistant bacterial colonization, but this relationship can be obscured in community settings owing to horizontal bacterial transmission and broad distributions. Locality-level exposure estimates considering inhabitants’ length of stay, exposure history, and exposure conditions of areas nearby could clarify these relationships. We used prescription data filled during 2010–2015 for 23 antibiotic types for members of georeferenced households in a population-based infectious disease surveillance platform. For each antibiotic and locality, we generated exposure estimates, expressed in defined daily doses (DDD) per 1000 inhabitant days of observation (IDO). We also estimated relevant environmental parameters, such as the distance of each locality to water, sanitation, and other amenities. We used data on ampicillin, ceftazidime, and trimethoprim-and-sulfamethoxazole resistant *Escherichia coli* colonization from stool cultures of asymptomatic individuals in randomly selected households. We tested exposure-colonization associations using permutation analysis of variance and logistic generalized linear mixed-effect models. Overall, exposure was highest for trimethoprim-sulfamethoxazole (1.8 DDD per 1000 IDO), followed by amoxicillin (0.7 DDD per 1000 IDO). Of 1,386 unique household samples from 195 locations tested between September 2015 and January 2016, 90%, 85% and 4% were colonized with *E*. *coli* resistant to trimethoprim and sulfamethoxazole, ampicillin, and ceftazidime, respectively. Ceftazidime-resistant *E*. *coli* colonization was common in areas with increased trimethoprim-sulfamethoxazole, cloxacillin, and erythromycin exposure. No association with any of the physical environmental variables was observed. We did not detect relationships between distribution patterns of ampicillin or trimethoprim-and-sulfamethoxazole resistant *E*. *coli* colonization and the risk factors assessed. Appropriate temporal and spatial scaling of raw antibiotic exposure data to account for evolution and ecological contexts of antibiotic resistance could clarify exposure-colonization relationships in community settings and inform community stewardship program.

## Introduction

Bacteria have evolved mechanisms by which to survive exposure to destructive or growth-inhibiting agents, a phenomenon referred to as antimicrobial resistance (AMR). Colonization by antimicrobial-resistant bacteria can indicate disturbance of host’s gut microbiome, which can impact its immune, neurological, and metabolic functions [1–3], resulting in poor health outcomes [4, 5]. Antimicrobial-resistant commensals can act as reservoirs for genetic elements that encode resistance, which, when shared with disease-causing bacteria, could result in bacterial infections that are difficult and expensive to treat [6, 7].

Antibiotics are important drivers of the emergence and persistence of resistant bacteria. With global use rates increasing [8, 9], including among outpatients [10], monitoring of populations at risk of carrying AMR bacteria is important, and can help to inform AMR control interventions. However, studies that have examined antibiotics use-resistance colonization relationship have yielded mixed results; observing associations in data rich regional- and national-level ecological designed studies [11–13], and some intercommunity and household-level studies [14, 15], but rarely in data-limited global-level studies [16–18] and intracommunity studies [19].

The failure to detect relationships at the community level may be attributable in part to mismatches between the spatiotemporal scales at which ecological and evolutionary processes that modulate AMR emergence and distribution occur, and the resolution at which exposure predictor variables are measured. Broad-scale horizontal movements of bacteria within community setting and persistence of resistant bacteria long after antibiotic use cessation [1, 14, 20] suggest that differentiation of bacteria strains on the basis of susceptibility to antibiotics in community setting could occur over broad time and spatial scales [21], likely following patches habitable to the different strains that may emerge owing to long-term patterns of antibiotic exposure.

In this study, we assess whether distributions of asymptomatic humans colonized with *E*. *coli* resistant to specific antibiotics are associated with gradients of specific antibiotic exposure derived from long-term (2010–2015), population-wide antibiotic use patterns. We used raster data layers summarizing exposure in terms of number of inhabitants, their length of stay in the locality, exposure history and exposure condition of localities nearby, to generate antibiotic exposure landscapes. This study represents our first major step toward an understanding of the environmental factors that are associated with appearance of resistant bacterial types.

## Methods

### Study setting

We performed the study in Kibera, a densely populated, informal urban settlement in Nairobi, Kenya. We extracted data from the Population-Based Infectious Disease Surveillance (PBIDS) platform, operated by the Kenya Medical Research Institute (KEMRI) in collaboration with the U.S. Centers for Disease Control and Prevention (CDC). The area under surveillance covered ~0.4 km^2^, and is characterized by inadequate sanitation, high infectious disease burden, high antibiotic use, and high prevalence of AMR colonization [19, 22]. In all, 11,432 households—a group of people residing in the same dwelling, eating from the same pot, and sharing common resources—participated in the surveillance program between 1 January 2010 and 31 August 2015. Of these households, 10,098 (88%) households were documented with global positioning system (GPS) coordinates. The median number of individuals per household was 6 (interquartile range 4–8).

### Household and clinic surveillance

The PBIDS methods have been described in considerable detail previously [22]. In brief, though, approximately 26,000 residents of all ages from approximately 5300 households were followed each year through household- and clinic-based surveillance. During the regular household surveys, community interviewers documented self- and proxy-reported demographic information (births, deaths, in-migrations, and out-migrations) and data on recent illness and healthcare seeking. Demographic data were used to estimate participants’ duration of participation in the study. PBIDS participants received medical care for acute infectious illness at no charge at a centrally located surveillance clinic (Tabitha clinic). Standardized clinical data, including antibiotic prescriptions filled, were collected at the clinic for participants meeting standardized case definitions [22]. Based on routinely collected household data, about 70% of participants reporting medically attended acute infections sought care at the surveillance clinic.

#### Data collection and preparation

*Proximity raster layers*. We used handheld Garmin, GPSMap62SC, and GPSMap76CSx geolocation units to collect geographic coordinates of water points, public toilets, dump sites, surface wastewater drainage networks, rivers, schools, clinics, and pharmacies across the study area. We extracted coordinates for the same set of features outside the study area from Map Kibera, an open digital map of the Kibera settlement [23]. Only features present between January 2010 and August 2015 were deemed influential to resistance distribution during the analysis period, and were included in the analyses. We converted points collected for wastewater drainage networks and rivers into polylines, and generated raster layers summarizing the distance from each locality to these features using the raster proximity analysis tool in QGIS (version 3.22.1). Distances were estimated across a 100 m buffer around the study area to account for features located outside the study area but close enough to be relevant to households located on the periphery of the surveillance area.

#### Antibiotic exposure raster layers

*1*. *Overview*. We extracted data on prescriptions filled for participants presenting with acute infections at the surveillance clinic between 1 January 2010 and 31 August 2015, and whose household GPS coordinates were available at the time of the clinic visit. In total, 104,814 prescriptions were filled, of which 33,409 (32%) were antibiotics. Antibiotic-related data extracted from the filled prescriptions included name, dose, daily units for consumption, and days of use. The filled prescription data were extracted on 12 February 2020. We estimated defined daily dosage (DDD) for each PBIDS household using World Health Organization (WHO) DDD recommendations [24]. We divided household DDD by the total days of observation of the household, to estimate person-time-of-observation normalized household exposure. Households that did not have any antibiotic exposure during the five-year period were considered to have zero exposure. To extend risk estimates across space, we interpolated among household exposure point data [25]. We expressed exposure estimates in the form of DDD per 1000 inhabitant-days of observation (IDO) and refer to these estimates as “exposure” from this point on; further methodological detail is provided below.

II. Estimating defined daily dosage.

a. *Antibiotic types*

We estimated DDD for every PBIDS participant with a filled antibiotic prescription between January 2010 and August 2015 following WHO’s DDD recommendations [24]. In brief, we estimated the total dosage as a product of antibiotic unit strength in grams, number of daily prescribed units, and number of prescribed days of use. These estimates were generated for each antibiotic. We estimated dosage for each time point and added these together for individuals that had multiple prescriptions filled during the study period. We then estimated DDD by dividing the estimated dosage with the WHO-Anatomical Therapeutic Chemical (ATC)-assigned DDD index unit for the specific antibiotic (WHO, version 2022) [26]. To obtain household-level DDDs, we summed DDDs for all household individuals; for overall DDDs, we summed all household DDDs.

For trimethoprim-sulfamethoxazole, we estimated DDDs as described above, assuming daily prophylactic use between the date of enrollment into a HIV comprehensive care (CC) program and the exit date (last program visit due to death or loss to follow-up), or a stop date of 31 August 2015. We used the participant’s age at the exit visit/stop date to estimate the strength of trimethoprim-sulfamethoxazole; for children aged 6 to 10 years, we used 0.48 g (480 mg), whereas for individuals ≥10 years old we used 0.96 g based on national guidelines for prophylactic antibiotic use [27]. Trimethoprim-sulfamethoxazole was rarely prescribed for treatment at the clinic, so we only included prophylactic use for individuals who were active in the CC program (receiving trimethoprim-sulfamethoxazole fills) in this analysis.

b. *Antibiotic groups*

To estimate DDDs for antibiotic groups, we first defined groups by similar chemical mechanisms of action [24, 26]. We grouped amoxicillin, amoxicillin-clavulanic acid, ampiclox, cloxacillin, benzathine penicillin, benzyl penicillin, flucloxacillin and phenoxy-methicillin as penicillins (J01C, [26]); ceftriaxone, cefuroxime, and cephalexin as cephalosporins (J01D); azithromycin, clarithromycin, and erythromycin as macrolides (J01FA); and ciprofloxacin, levofloxacin, nalidixic acid, and norfloxacin as quinolones (J01M). DDDs were estimated only with antibiotics used for treatment. We summed the estimated DDDs of each member of a group [24]. For example, for macrolides DDD, we summed the individual DDDs of azithromycin, clarithromycin, and erythromycin. We generated household-level and overall antibiotic group DDDs as described for the antibiotic types above.

#### III. Estimating days of observation

We calculated total days of household participation in PBIDS (i.e., time a household had at least one PBIDS participant and therefore eligible to receive a prescription from the study clinic) as the sum of days of participation of all PBIDS members of the household during the period January 2010 to August 2015. Households with more PBIDS members therefore contributed more household-time. For overall days of participation, we summed days of participation across all households. To be eligible to participate in the surveillance, individuals had to have resided in the study area for at least 4 months (or be born to a PBIDS participant) [22]. PBIDS participants contributed person-time during times of residency, and stopped if they moved from the study area for more than four consecutive months.

*II*. *Estimating antibiotic exposure raster layers*. We estimated overall exposure for each antibiotic type and group by dividing overall DDDs by the overall days of participation. For household-level exposure, we divided a household’s total DDDs by its total days of observation. To account for precision in exposure estimates between households, we constructed raster layers by interpolation of household level risks using the cubic spline method in QGIS (version 3.22.1) [25].

#### Population density raster layer

To generate a population density raster layer, we estimated daily household sizes by dividing cumulative household sizes by the households’ time of observation. Household size referred to the number of persons that lived in a PBIDS household. Using the rasterize tool in QGIS (version 3.22.1) [25], we assigned to each raster cell the estimated daily household size value for the PBIDS household at that location.

### Antibiotic resistance colonization

We used data on resistant *E*. *coli* colonization in asymptomatic individuals from a previous longitudinal study conducted in the same PBIDS population. Sample collection and processing methods have been described elsewhere [19]. Briefly, however, 200 households were selected randomly, each from among households with at least one adult (≥18 years old) and one child (≤5 years old) from each housing block across the study area for demographic and spatial representation. Households selected were distributed across 195 unique locations, as defined by their geocoordinates. Households shared the same geocoordinates if they shared a roof and entrance point.

Households were sampled approximately every two weeks between September 2015 and January 2016. Two stool samples were collected (from one adult ≥18 years, and from one child <5 years of age, when available) per household. A gram of stool from each sample was emulsified in phosphate-buffered saline, serially diluted, and then plated on MacConkey agar using sterile glass beads. Twelve presumptive *E*. *coli* (24 for two samples per household) were selected randomly from each plate, and sub-cultured on nutrient broth in a 96-well plate. A 96‐well pin replicator was then used to transfer isolates onto 150 mm MacConkey agar plates, each with one of six antibiotics (32 μg/mL ampicillin; 8 μg/mL ceftazidime; 32 μg/mL chloramphenicol; 4 μg/mL ciprofloxacin; 64 μg/mL sulfamethoxazole; and 16 μg/ml trimethoprim, all from Sigma, St. Louis, MO). A plate without antibiotics was included to confirm cell viability. Isolates were scored as resistant (complete growth) or susceptible (no growth or partial growth). The proportion of resistant *E*. *coli* per gram of stool in a household was estimated by dividing the total number of specific resistant *E*. *coli* colonies identified from persons tested at the household by the total number of colonies tested for resistance at the household. Direct plating of serially diluted stool onto nonselective medium, aimed at detecting actively colonizing resistant *E*. *coli* strains which we assumed reflected selection *in situ*.

#### Data organization

We classified household samples collected <14 days apart from the same location as repeat samples, and combined them by dividing the total number of resistant *E*. *coli* identified from households in the location by the total isolates tested for antibiotic susceptibility in the households in that location [19]. We transformed the resistance data into presence-absence (1, 0) for the pathogen niche modelling framework. To account for potential testing errors, we considered a household as “colonized” if ≥0.25 of its total isolates were resistant to an antibiotic. For analyses in this contribution, we used ampicillin, ceftazidime, trimethoprim and sulfamethoxazole resistant *E*. *coli* colonization data. Ceftazidime resistant *E*. *coli* colonization was rare, whereas colonization with the other three antibiotics was common in the area. Since resistance to trimethoprim and sulfamethoxazole often occurs together [28], we considered colonization with *E*. *coli* resistant to trimethoprim and sulfamethoxazole where tested *E*. *coli* were resistant to both antibiotics, and susceptible where tested *E*. *coli* was susceptible to both or either of the antibiotics.

We transformed all environmental predictor raster layers to the same spatial resolution (2 x 2 m) and spatial extent (1516 x 476 m) using raster aggregation (average of values) procedures; we used one antibiotic exposure raster layer as a reference to which pixels of all other raster layers were matched at each resolution using nearest neighbor-aggregation method in package raster, version 3.6–26, in R, version 4.2.2 [29, 30]. For each coordinate of households tested for antibiotic resistant *E*. *coli* colonization, we identified the raster cell containing the coordinate, and assigned estimated values for antibiotic exposure, distance, and population density of the cell to the coordinate.

Antibiotic resistant gut commensals selected by antibiotics exposure can persist for periods longer than six months following termination of antibiotic use by individuals in community settings [20]. This turnover lag period in the gut microbial community is also observed broadly in populations [1]. For this analysis, we therefore used exposure estimates for the period 1 January 2010 and 31 August 2015 to predict resistance outcomes from repeat testing during the period 1 September 2015 and 22 January 2016.

#### Data analyses

We summarized overall antibiotic exposure conditions in the study area by type and group, sampling frequency, distribution of sampled locations across geographic and environmental spaces of the study area [31], and the distribution of sampled households by antibiotic exposure conditions of their locations. We tested for collinearity between predictor variables using a Pearson correlation coefficient (*r*) threshold of >0.7. For comparison, we used two modeling approaches to assess and evaluate factors potentially associated with occurrence of individuals carrying resistant *E*. *coli*.

- **Permutational Multivariate Analysis of Variance**
  We used PERMANOVA to contrast environmental conditions (state of antibiotic exposure and other factors assessed at a locality) of households with individuals with resistant *E*. *coli* against null conditions. The latter were derived from 999 random samples derived from the pool of all households sampled [32, 33]. Mahalanobis distance was used as the measure of variance. We estimated centroids (i.e., mean values of the coordinates) of risk factors assessed for colonized households. We randomly drew 999 subsamples of size matching the observed number of colonized households from the pool of colonized and uncolonized households, and estimated centroids for the risk factors for each subsample to generate a null distribution. We established whether centroids of colonized households fell within the 95% quantiles of the null distribution, and considered centroids that fell outside this distribution as different from the null for the assessed factors. We then performed univariate analyses of variable sets in which we detected significant signals of dissimilarity to identify variables independently associated with resistance colonization, and to characterize whether observed centroids fell at the lower or upper end of the null distribution. PERMANOVA models were generated with the vegan package [34]. Statistical significance was defined based on *α* =  0.05.
- **Mixed-effects logistic regression**
  We fitted a logistic generalized linear mixed-effect model using sampling locality (latitude and longitude) as the random variable to account for variation owing to repeat sampling. We standardized each predictor variable by subtracting respective means and dividing by the standard deviation. We then calculated adjusted odds ratios (aORs). Models were generated using the package lme4 version 1.1–34 [35].

To avoid collinearity between antibiotic types and groups, which were generated as sums of antibiotic types, we fit models using two separate datasets: one with antibiotic types, and another with groups as predictors. To account for potential effects of migration, we tested for associations using complete datasets and datasets excluding households with reported migration in the twelve months prior to sampling.

### Ethics statement

Ethical approvals for the studies from which data were drawn were obtained from the KEMRI Scientific and Ethics Review Committee (SSC protocol numbers 3928, 2998, 1899 & 2761) and the US CDC Institutional Review Board (protocol numbers 4566 & 6775). Participation in each study was voluntary, and participants could decline participation at any time. For PBIDS, written informed consent was obtained from heads of households for their household members to participate; individual household members could decline participation in the surveillance. For the carriage study, additional written informed consent was obtained from household heads and participating adults prior to study enrollment. For children aged <7 years, parental consent was provided by the parent or guardian; for children aged 7–17 years, parental consent and a written assent from the child were obtained. All data analyzed were anonymized to ensure confidentiality of study participants.

## Results

**Fig 1:**
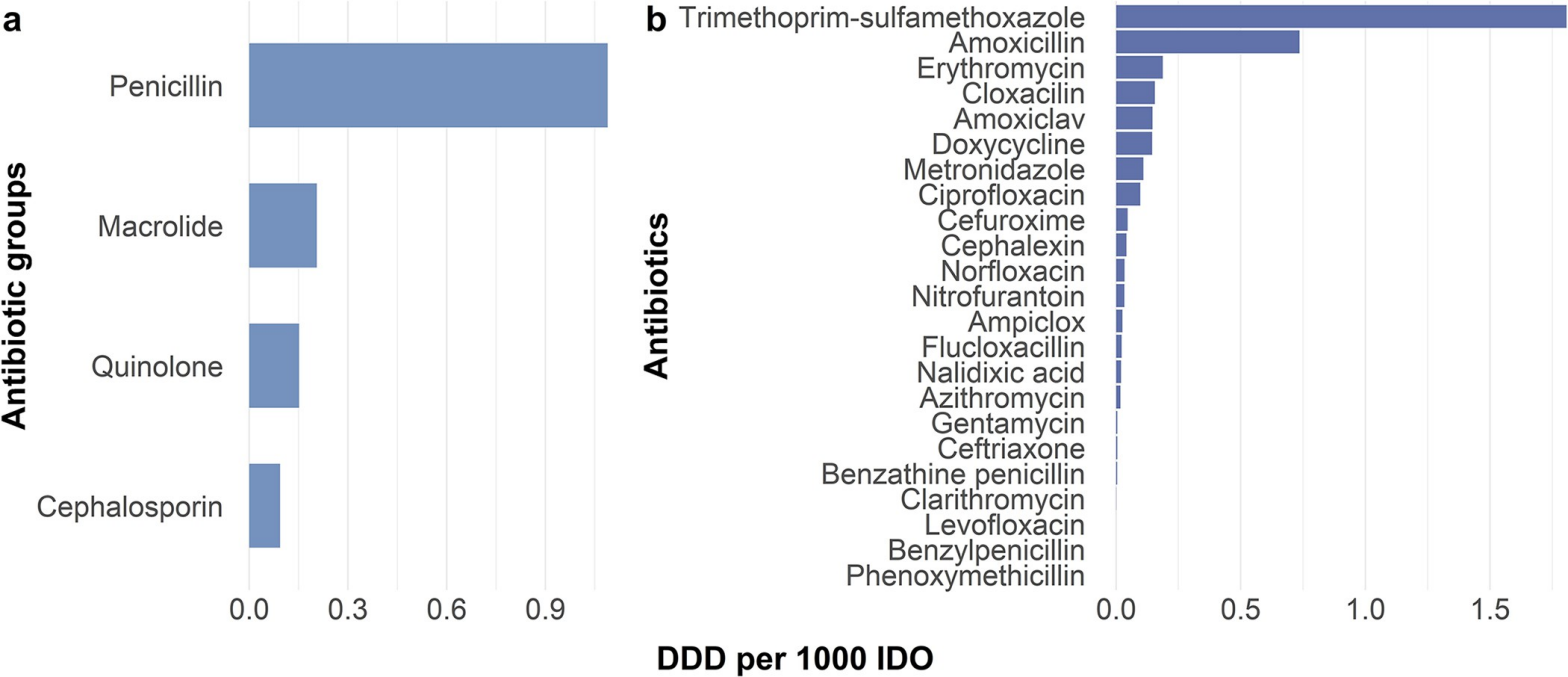
Overall antibiotic exposure estimates by group (a) and type (b). Exposure estimates expressed in numbers of defined daily dosage (DDD) per 1000 inhabitant-days of observation (IDO). Antibiotic exposure estimates were calculated using prescription fills data obtained from a Kibera population-based infectious diseases surveillance platform during the period January 2010 –August 2015. DDDs were estimated using WHO’s DDD guidelines. Estimated total DDD for each antibiotic type was divided by total days of observation of all households that participated in the surveillance during the same period (57,778,396 household-days of observation). DDDs for specific antibiotic groups were calculated by summing DDDs of members of a group. Group members were defined based on similarity in their mechanisms of actions. Generated group DDDs were divided into the total days of observation of all households in the surveillance.

During 1 January 2010 and 31 August 2015, 10,098 households in 8285 unique locations were monitored, for a total of 57,778,869 household days. Of these households, 5089 (50.4%) households at 4878 locations had at least one person exposed to one of the antibiotics assessed. Overall estimated exposure across the study population ranged from 0.1 DDD per 1000 IDO among cephalosporins to 1.1 DDD per 1000 IDO among penicillins for antibiotic group (Fig 1), and from 7 x 10^−6^ DDD per 1000 IDO for phenoxymethicillin to 1.8 DDD per 1000 IDO for trimethoprim-sulfamethoxazole for antibiotic types.

**Fig 2:**
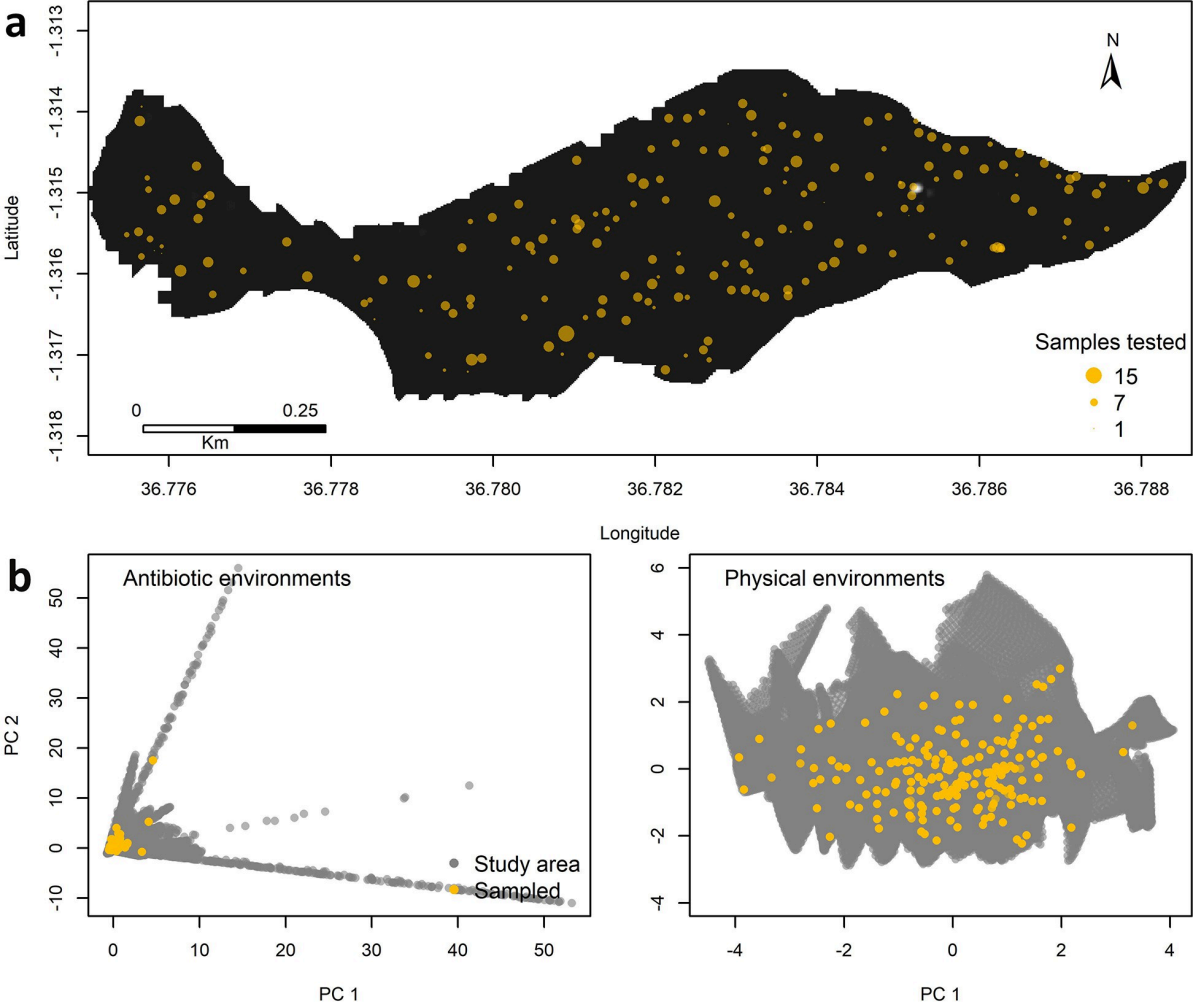
Distribution of sample testing in geographic space (a), and distribution of localities sampled in environmental space (b). The map and yellow dots show distribution and number of household samples tested across the geographic area of the Kibera population-based infectious diseases surveillance platform. The size of the dots represents numbers of samples tested during the period September 2015 –January 2016. The plots show the first principal components (PC) of 23 antibiotic variables (antibiotic environments) and variables summarizing distances to the select geographic features in the area (physical environments). Each of the dots (gray and yellow) represents an existing combination of principle components, that is, an existing, unique, environment in the study area. The yellow dots represent the environments of location of households sampled i.e., those that were tested for resistance.

In total, 1386 unique household samples from 195 locations were tested between 1 September 2015 and 22 January 2016. Of these samples, 1341 (97%) from 191 unique locations linked with all environmental raster layers and were included in the final analysis; 1163 (87%) samples were from households with no migration history in the 12 months prior to the start of sampling. The mean number of household samples tested per location surveyed was 7 (range 1–15) (Fig 2A). The localities sampled were distributed homogenously across the study area. However, in the environmental space, these locations did not capture all of the extremes of environmental conditions for antibiotic exposure and physical environments (Fig 2B).

**Fig 3:**
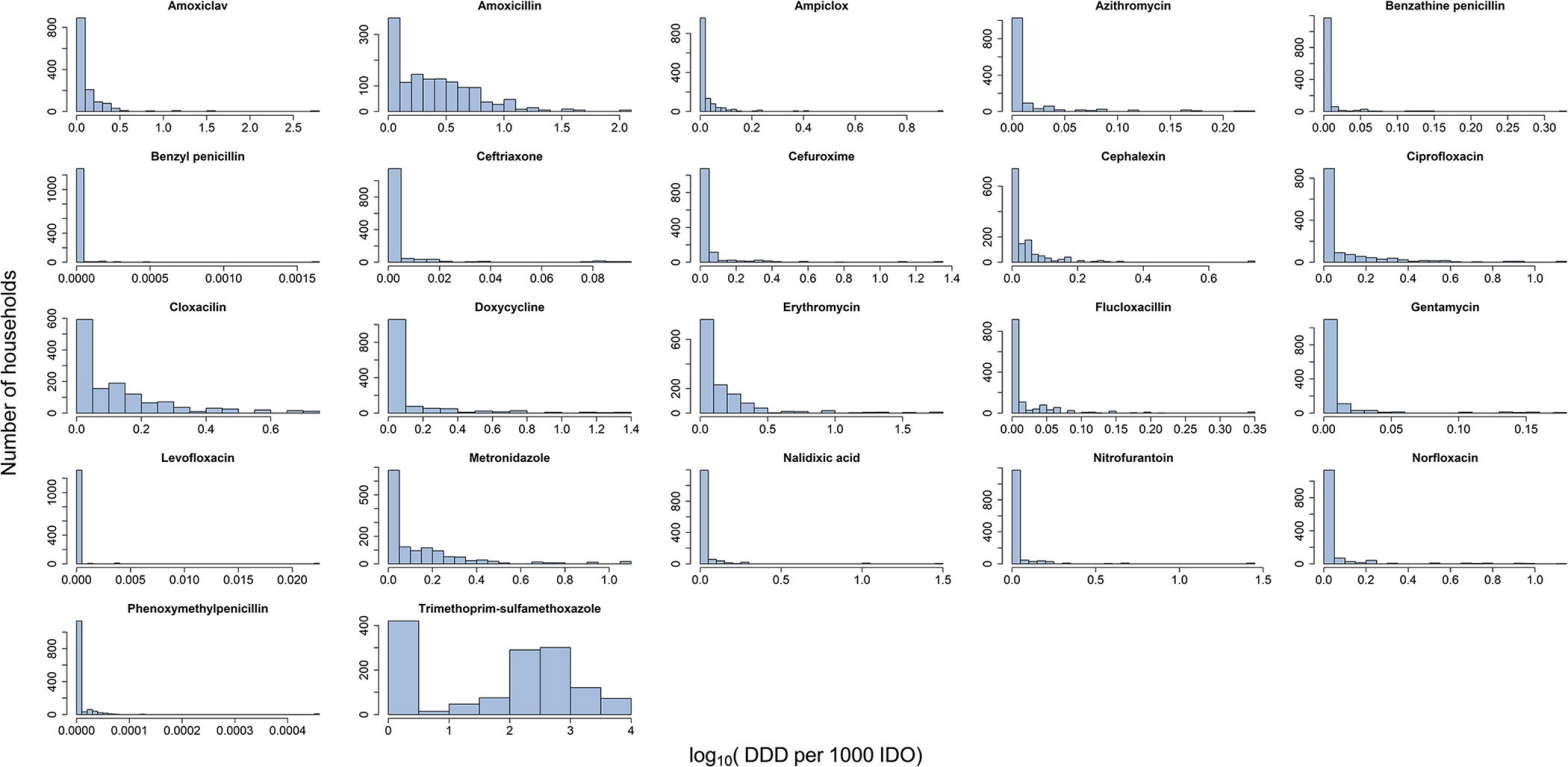
Distribution of households sampled across exposure levels of antibiotics evaluated. Bars represent counts of households. Exposure is shown as log_10_-transformed numbers of defined daily dosage (DDD) per 1000 inhabitant-days of observation (IDO). To estimate locational antibiotic exposure, household-level exposure, estimated by dividing a household total DDDs by its total days of observation, were interpolated using cubic spline interpolation in QGIS.

When we analyzed the distribution of the sampled households by antibiotic exposure conditions of their localities, households in locations with amoxicillin exposure were most common (1072; 80%), followed by cloxacillin exposure (1010; 75%) (Table 1). Fewer households were in areas with benzyl penicillin (77, 6%), levofloxacin (59, 4%), or clarithromycin (4, <1%) exposure. Nitrofurantoin and benzyl penicillin were highly correlated (*r* = 0.9), so we excluded benzyl penicillin, which was used less commonly. We also excluded clarithromycin from multivariate analysis because it was rarely prescribed among the study population. Exposure levels for antibiotics evaluated varied considerably across localities of the households sampled (Fig 3).

**Table 1:** Distribution of households sampled (*n* = 1341) by antibiotic exposure type in their localities (January 2010 –August 2015).

Antibiotics	No. of households *n (%)*
Amoxicillin	1072 (79.9)
Cloxacillin	1010 (75.3)
Metronidazole	975 (72.7)
Erythromycin	946 (70.5)
Trimethoprim-sulfamethoxazole	921 (68.7)
Cephalexin	904 (67.4)
Amoxiclav	878 (65.5)
Ciprofloxacin	814 (60.7)
Gentamycin	770 (57.4)
Ampiclox	760 (56.7)
Benzathine penicillin	721 (53.8)
Flucloxacillin	677 (50.5)
Norfloxacin	627 (46.8)
Cefuroxime	600 (44.7)
Doxycycline	562 (41.9)
Azithromycin	559 (41.7)
Nalidixic acid	525 (39.1)
Phenoxymethylpenicillin	431 (32.1)
Ceftriaxone	414 (30.9)
Nitrofurantoin	358 (26.7)
Benzyl penicillin	77 (5.7)
Levofloxacin	59 (4.4)
Clarithromycin	4 (0.3)

**Fig 4:**
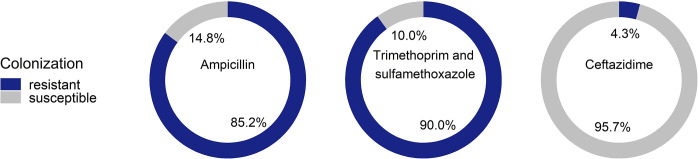
Proportion of samples colonized with *Escherichia coli* resistant to ampicillin, trimethoprim and sulfamethoxazole, and ceftazidime. Proportions calculated using total number of household samples tested (*n* = 1386).

The proportion of antibiotic resistant *E*. *coli* colonization varied widely. Of the samples tested, 90%, 85% and 4% were colonized with *E*. *coli* resistant to trimethoprim and sulfamethoxazole, ampicillin, and ceftazidime, respectively (Fig 4).

**Fig 5:**
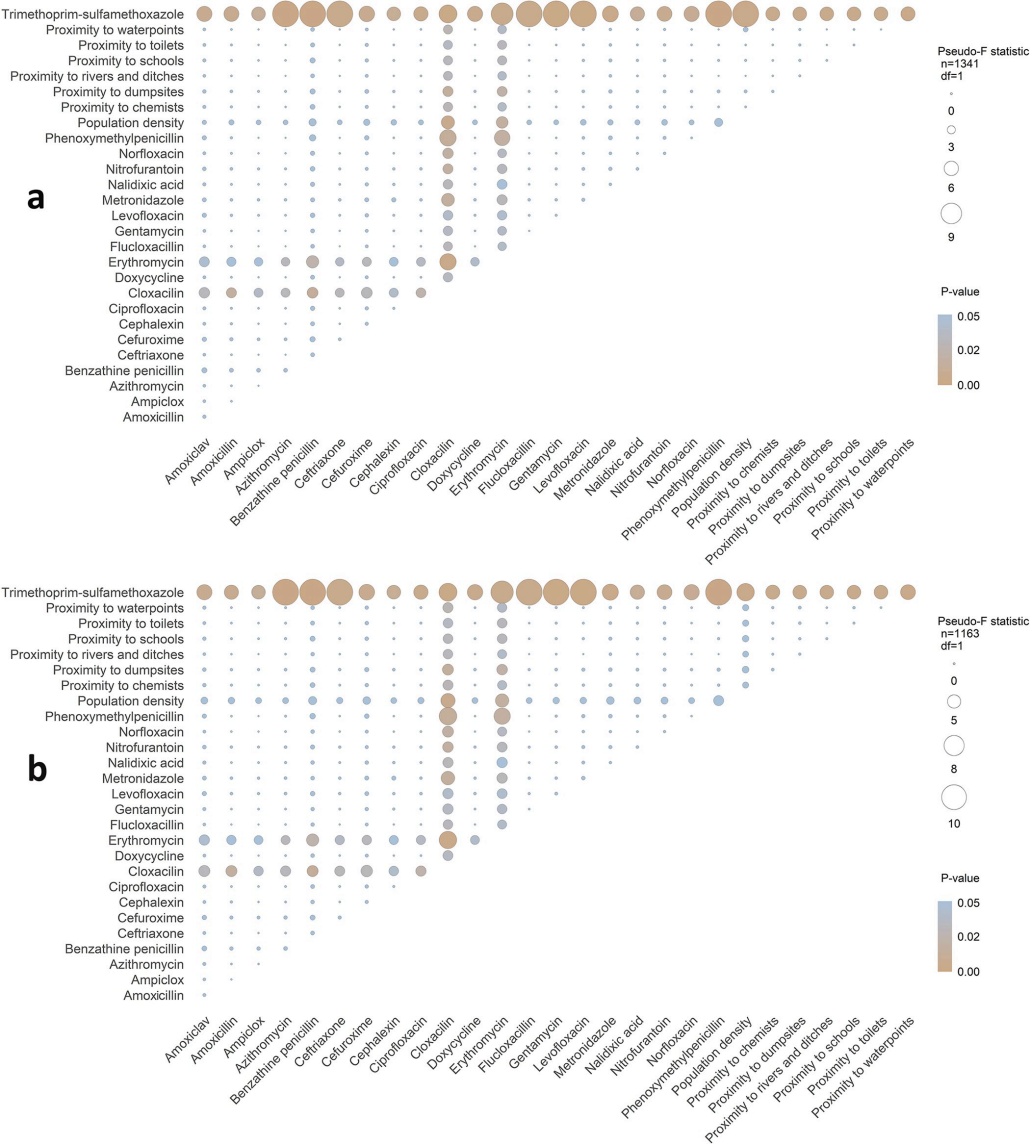
Heatmap of pairwise permutation analysis of variance (PERMANOVA) of antibiotic type and other environmental characteristics, for ceftazidime-resistant *E*. *coli*. Figure (a) represents results of analysis with complete data (n = 1341) whereas (b) represents analysis with data excluding households with reported migrations twelve months prior sample collections (n = 1163). Each bubble represents statistics from comparison of the paired conditions between areas where individuals carrying specific resistance were observed and null conditions derived by random subsamples (n = 999) of equal size to the number of positive households, drawn from all households tested for the resistance. Pseudo-*F* statistics are represented with sizes of the circle and *p*-values with color.

**Fig 6:**
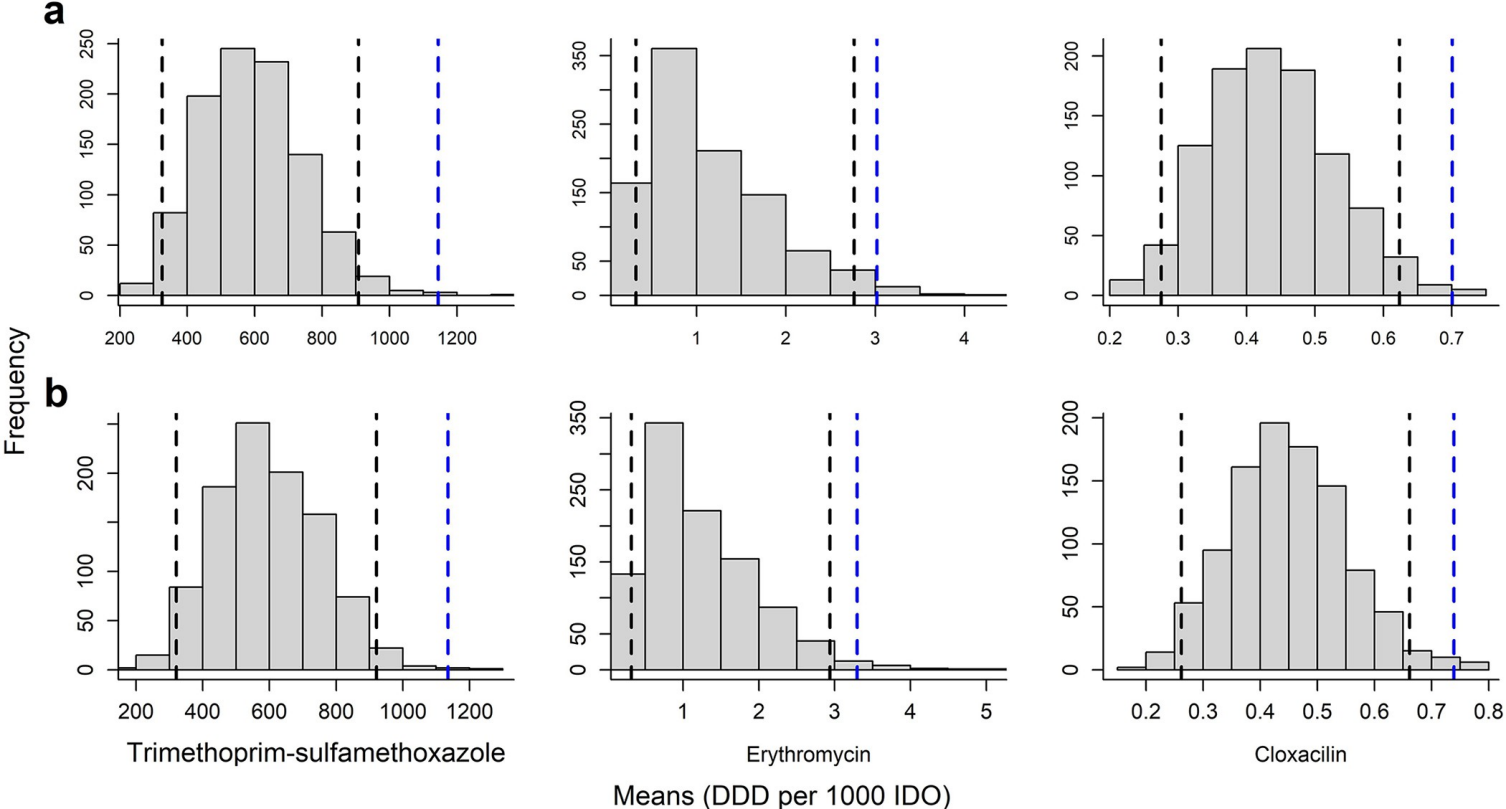
Univariate analysis comparing mean exposure (expressed as DDD per 1000 IDO) between areas where ceftazidime-resistant *Escherichia coli* occurred (blue dashed line) and null distribution of mean exposures derived from random subsamples (*n* = 999) of equal size to the number of positive households, drawn from all households tested for the resistance (bars). Black dashed line represents the 95% confidence levels. Figure (a) represents results of analysis with complete data (*n* = 1341) whereas (b) represents analysis with data excluding households with reported migrations twelve months prior sample collections (*n* = 1163).

Of the 378 pairs of antibiotic types and 55 pairs of antibiotic groups analyzed using PERMANOVA, exposure conditions for 75 pairs of antibiotic types differed significantly from null expectations in areas where individuals with ceftazidime-resistant *E*. *coli* strains were present (Fig 5A). No significant differences from null conditions were observed in areas where individuals carrying ampicillin, and trimethoprim and sulfamethoxazole resistant *E*. *coli* strains were present. Similarly, no significant departures from null expectations were observed across antibiotic groups. Univariate analyses identified trimethoprim-sulfamethoxazole [null exposure mean 589.0 DDD per 1000 IDO (95% CI: 325.0–908.0) vs mean exposure of cases colonized 1144.0 DDD per 1000 IDO], cloxacillin [0.4 DDD per 1000 IDO (95% CI: 0.3–0.6) vs 0.7 DDD per 1000 IDO], and erythromycin [1.1 DDD per 1000 IDO (95% CI: 0.3–2.8) vs 3.0 DDD per 1000 IDO] as factors non-randomly associated with ceftazidime-resistant *E*. *coli* colonization in the population (Fig 6A).

**Fig 7:**
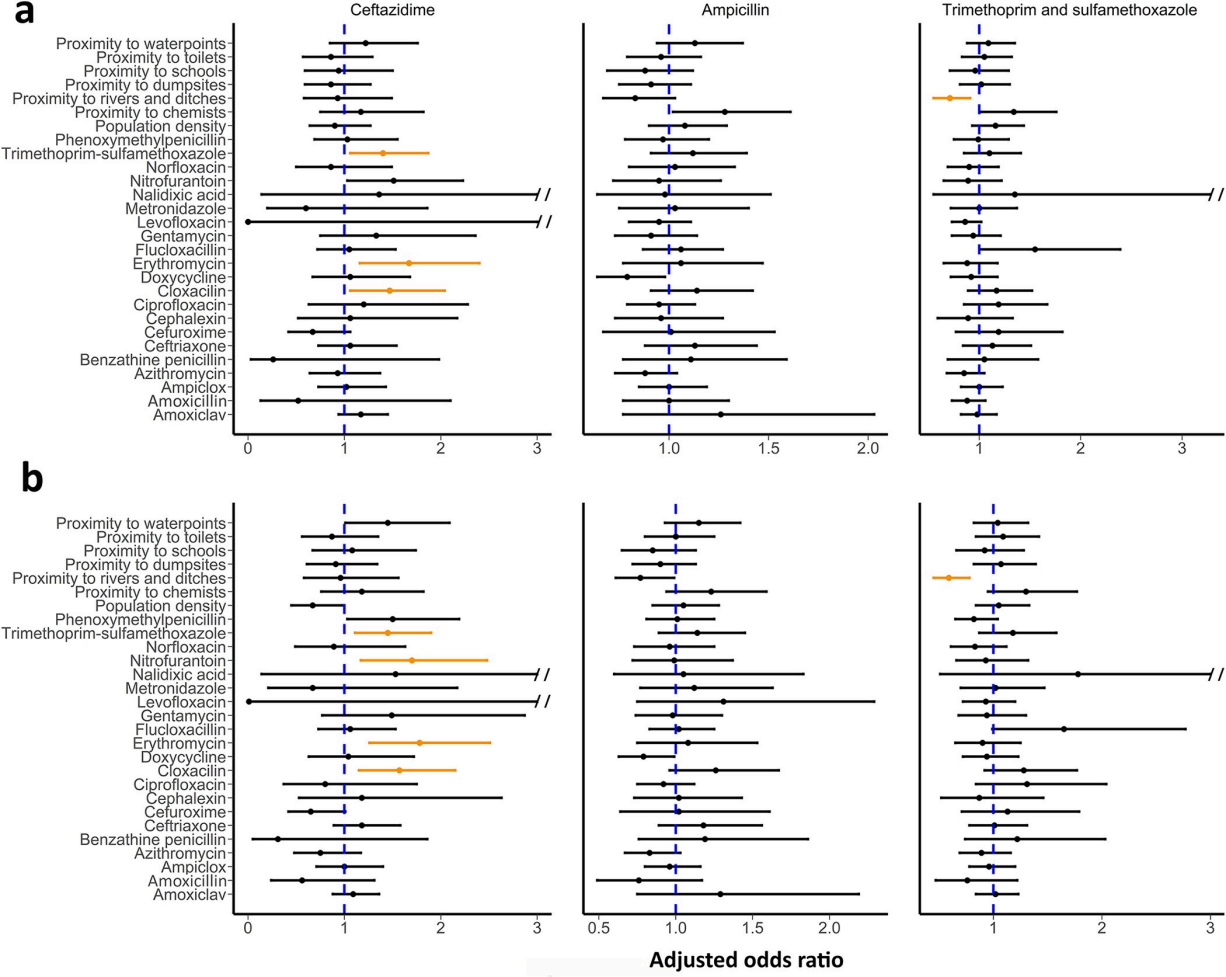
Adjusted odds ratios for ceftazidime, ampicillin, and trimethoprim and sulfamethoxazole resistant *Escherichia coli* colonization by antibiotic exposure, distance, and population density risk factors. Adjusted odds ratios were generated using logistic generalized linear mixed-effect models with sampling location as a random variable. Statistically significant factors are highlighted in orange. Double slash indicates break of upper error bar for non-statistically significant factors. Figure (a) represents results of analysis with complete data (*n* = 1341) whereas (b) represents analysis with data excluding households with reported migrations twelve months prior sample collections (*n* = 1163).

Similar results were observed using logistic generalized linear mixed-effect models: Ceftazidime-resistant *E*. *coli* colonization was associated with trimethoprim-sulfamethoxazole exposure [adjusted odds ratio (aOR) 1.4 (95% CI: 1.1–1.9)], cloxacillin exposure [aOR 1.5 (CI: 1.1–2.0)], and erythromycin exposure [aOR 1.7 (CI: 1.2–2.4)] (Fig 7A). We also observed association between proximity to rivers and ditches [aOR 0.7 (CI: 0.6–0.9)] with trimethoprim and sulfamethoxazole-resistant *E*. *coli* colonization. Observed relationships were maintained in analyses of the dataset excluding households with reported migrations 12 months prior to sampling (Figs 5B–7B). We observed an additional association between ceftazidime-resistant *E*. *coli* colonization and nitrofurantoin exposure [aOR 1.7 (CI: 1.2–2.5)] when we analyzed the data using logistic generalized linear mixed-effect models.

## Discussion

We observed consistent associations between ceftazidime-resistant *E*. *coli* colonization and areas of high trimethoprim-sulfamethoxazole, erythromycin, and cloxacillin exposure across the different analysis approaches used. However, these exposure conditions explained variation in the spatial distribution of resistance colonization risk only subtly and diffusely, which may reflect fluidity in carriage in densely populated settings with limited barriers to horizontal bacterial transmission. Similar to what was previously described in this setting [19], we did not observe differences between null exposure conditions and conditions in areas where ampicillin and trimethoprim and sulfamethoxazole-resistant *E*. *coli* colonization occurred, likely owing to ubiquity of these resistant strains in the area. Proximity to sanitation features and population density did not differ from random for areas where resistant *E*. *coli* colonization was observed.

Our results demonstrate that colonization with non-widespread resistant *E*. *coli* strain may follow gradients of specific antibiotic exposure in community settings. Associations between observed distributions and exposure estimates derived from long-term, population wide antibiotics exposure indicate that colonization distributions likely organize over long periods of time and at scales broader than individual. Studies examining exposure-colonization relationship may benefit from using locality-level estimates, with the size of locality informed by environment shared and physical interaction, that consider the number of inhabitants, time of stay, and exposure history, and which are adjusted for exposure conditions in localities nearby.

Few studies to date have observed and assessed exposure-colonization relationships in community settings, potentially owing to challenges in characterization of exposure [14, 19, 36, 37]. In estimating exposure using population-wide, long-term person-time-inclusive data, and constructing risk gradients through collating sporadic antibiotics exposure across space, we improved characterization of underlying, potentially stable, gradients of selective forces potentially constraining the distribution of resistant *E*. *coli* colonization in this population [38]. The approach is historically and spatially contingent, and takes to account the idea that individuals reporting no recent antibiotic exposure may carry resistant bacteria if residing in or near areas with individuals with history of exposure and *vice versa* [39–41]. The pathogen niche assessment framework that we employed [32] augments assessment of exposure-colonization relationships in populations by accounting for potential nonnormality in distributions of resistant bacteria colonization phenomenon, background resistance patterns [42], and biases in sampling with respect to environments. Repeat measurement of resistance during the five study months likely increased the chances for observing truer distribution of resistant *E*. *coli* colonization in the community.

The associations that we documented between ceftazidime-resistant *E*. *coli* colonization and high cloxacillin exposure conditions are consistent with adaptation to beta-lactam antibiotics [43]. Inhibition of bactericidal effects of beta-lactams by erythromycin [44] likely facilitates bacterial adaptation to beta-lactams, whereas potential upregulation of genes encoding beta-lactamases when co-occurring with trimethoprim-sulfamethoxazole or nitrofurantoin resistant genes [45–47] could explain trimethoprim-sulfamethoxazole and nitrofurantoin associations with ceftazidime-resistant *E*. *coli* colonization. We did not observe associations between colonization with *E*. *coli* resistant to trimethoprim and sulfamethoxazole or ampicillin and assessed antibiotic exposure predictors. Strains resistant to these antibiotics were widely prevalent in the population, suggesting large-scale displacement of susceptible *E*. *coli*. Such ubiquity of resistant strains favors horizontal movement of resistant bacteria, including among individuals not exposed to antibiotics [40], which can impact the statistical power of the tests that we used and in effect limit ability to detect real, existing relationships. The risk of trimethoprim and sulfamethoxazole resistant *E*. *coli* colonization increased the closer that individuals lived to rivers and ditches draining the area, suggesting that these features may serve as dispersers of bacteria, including resistant variants in the area.

These findings are reported with the following limitations. We did not consider exposure information of antibiotics received for clinical conditions not meeting surveillance definition of acute infection, or from other non-study pharmacies in the area, which could have underestimated the exposure. A previous study in the area observed higher proportions of reported antibiotics exposure (70–87%) [48]. We also assumed that prescriptions being filled translated into actual use, even though not everyone who receives an antibiotic uses it, nor do all users complete their antibiotic dose recommendations. Our microbiological procedures, though optimized for detection of selection occurring in natural settings, may have underestimated the true burden and potential distribution of resistant *E*. *coli* colonization in the area. A recent study in the same population that used different testing approaches found that about half of the population carried extended-spectrum cephalosporin-resistant Enterobacteriaceae [49]. However, selecting, at the laboratory level, for resistant strains that would otherwise be crowded out by susceptible strains may indicate cases of unsuccessful dispersal (i.e., dispersal that did not lead to colonization). A further concern is that a relatively small, highly interactive area was studied, which may limit clear delineation of distribution patterns that might be evident across a broader area. Although we used exposure layers generated with antibiotic data normalized over a period of 5 years, an appropriate time scale has not been tested.

We found relationships between exposure gradients of commonly used antibiotics, including trimethoprim-sulfamethoxazole, which is used as prophylactic antibiotic among HIV-infected persons, and colonization distribution of ceftazidime-resistant *E*. *coli*. Considering these antibiotics in community stewardship programs could reduce ceftazidime resistance colonization. Associations were complex, involving co-selection and potential expression modification of resistance genes, highlighting the importance of exploring a wide range of antibiotics when assessing exposure-colonization relationships in populations. Overall, appropriate scaling of raw exposure data to account for historical and spatial dependencies, could clarify relationships between antibiotic exposure and colonization in community setting. Better characterization of colonization-exposure relationships could be achieved with analyses considering more diverse exposure environments, across larger geographic areas.
